# M48U1 CD4 mimetic has a sustained inhibitory effect on cell-associated HIV-1 by attenuating virion infectivity through gp120 shedding

**DOI:** 10.1186/1742-4690-10-12

**Published:** 2013-02-01

**Authors:** Philippe Selhorst, Katrijn Grupping, Tommy Tong, Ema T Crooks, Loïc Martin, Guido Vanham, James M Binley, Kevin K Ariën

**Affiliations:** 1 Department of Biomedical Sciences, Unit of Virology, Institute of Tropical Medicine, Antwerp, B-2000, Belgium; 2 Torrey Pines Institute for Molecular Studies, San Diego, CA 92121, USA; 3 Commissariat à l'énergie atomique et aux énergies alternatives, iBiTecS, SIMOPRO, Gif sur Yvette, F-91191, France; 4 Faculty of Pharmaceutical, Biomedical and Veterinary Sciences, University of Antwerp, Antwerp, B-2000, Belgium

**Keywords:** HIV, Entry, CD4 binding site, gp120, Shedding, Inhibition

## Abstract

**Background:**

HIV-1 infected cells can establish new infections by crossing the vaginal epithelia and subsequently producing virus in a milieu that avoids the high microbicide concentrations of the vaginal lumen.

**Findings:**

To address this problem, here, we report that pretreatment of HIV-infected peripheral blood mononuclear cells (PBMCs) with a 27 amino acid CD4-mimetic, M48U1, causes dramatic and prolonged reduction of infectious virus output, due to its induction of gp120 shedding.

**Conclusions:**

M48U1 may, therefore, be valuable for prophylaxis of mucosal HIV-1 transmission.

## Findings

**Figure 1:**
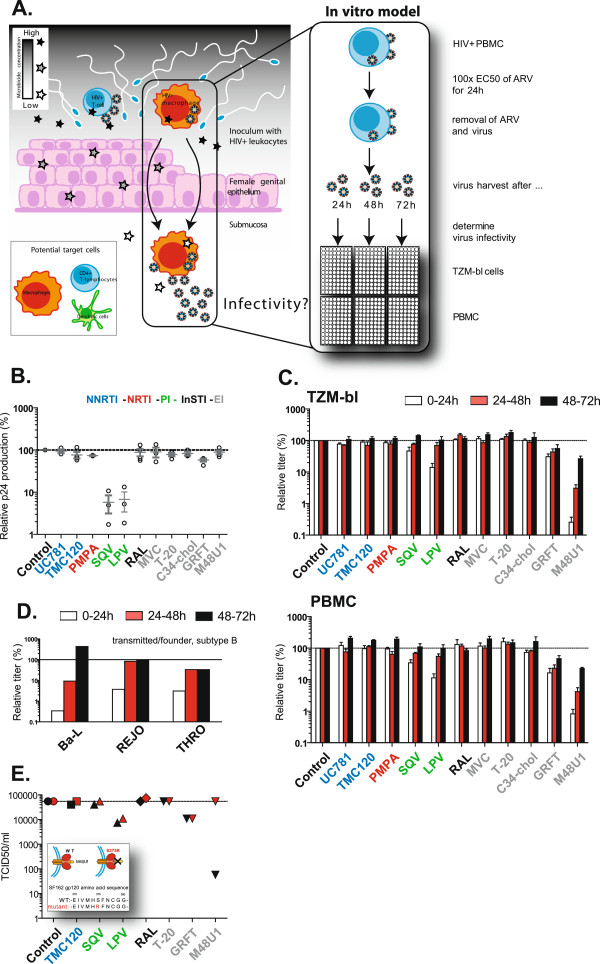
**M48U1 has a memory effect on cell-associated virus. A)** The Trojan Horse transmission concept was modeled *in vitro* using HIV-infected PBMCs which were treated for 24 h with antiretrovirals (ARVs) from different classes at 100x EC50 concentrations. Subsequently, virus production was quantified using a Gag p24 ELISA. To mimic escape from vaginal drug pressure, the infected cells were then incubated in ARV-free medium for three consecutive periods of 24 h. Infectivity of newly produced virions was determined after each period by titration in TZM-bl cells and PBMCs using equal amounts of p24. **B)** Production of Gag p24 by infected PBMCs during 24 h treatment with eleven ARVs: the non-nucleoside reverse transcriptase inhibitors (NNRTIs; blue) UC781 and TMC120, the nucleotide reverse transcriptase inhibitor tenofovir (PMPA; red), the entry inhibitors (grey) maraviroc (MVC), T-20, C34-chol, griffithsin (GRFT) and M48U1, the protease inhibitors (green) lopinavir (LPV) and saquinavir (SQV), and the integrase inhibitor raltegravir (RAL). Values are expressed relative to untreated control cultures and represent the mean +/− SEM of at least three independent experiments, each carried out in triplicate. **C)** Bal infectivity of *de novo* produced virus by pretreated infected PBMCs in absence of drug during three consecutive periods of 24 h. Values are expressed relative to untreated control cultures and represent the mean +/− SEM of at least three independent experiments, each carried out in triplicate. **D)** Infectivity of *de novo* produced transmitted/founder viruses REJO and THRO (subtype B) and Bal by M48U1 treated infected PBMCs in absence of drug during three consecutive periods of 24 h. Values are expressed relative to untreated control cultures. **E)** Infectivity of wild type (black) and M48U1-resistant (red) Bal virus produced during a 24 h period in absence of drug by pretreated infected PBMCs. Values represent the 50% Tissue Culture Infective Dose (TCID50)/ml as measured in one experiment carried out in triplicate.

The majority of new HIV infections worldwide are acquired through heterosexual transmission. Although receptive transmission at the vaginal mucosa is thought to be primarily caused by cell-free virus (CFV) [1], it may also involve transfer of HIV-infected leucocytes (i.e., cell-associated virus; CAV) present in semen [2,3]. The observation that CFV infection via the vaginal mucosa requires a ~10^3^-10^6^ higher virus dose [4] than is needed to establish infection by the intravenous route suggests that the healthy epithelium of the female genital tract is a robust barrier to HIV transmission. However, in contrast to CFV, infected seminal lymphocytes or macrophages are capable of migrating through intact epithelia and delivering virus directly to the submucosa or even the draining lymph nodes. This putative ‘Trojan Horse’ infection route (Figure 1A) is supported by various studies in mouse and macaque models [4-9].

Vaginal microbicides currently under development to prevent heterosexual HIV transmission should, therefore, ideally be able to inactivate virus in these migrating leucocytes, as well as CFV. Although most candidate microbicides, including entry inhibitors, can inhibit CAV *in vitro*[10-12], their activity *in vivo* depends on the inhibitor concentrations that can be achieved in the cervical and vaginal (sub) mucosa where most of the target cells reside. A study in rabbits and macaques measured the levels of the candidate microbicide dapivirine (TMC120) in the cervicovaginal tissue and found that drug-related material was primarily detected in the superficial cellular layers of the mucosal epithelia and not in the submucosa or draining lymph nodes [13]. Hence, following the proposed CAV or Trojan Horse concept (Figure 1A), infected seminal leucocytes could subvert drug pressure in the vaginal lumen by migrating to the submucosa or regional lymph nodes. Subsequently, infection may be established by virions budding from these migrating leucocytes. However, it remains possible that initial vaginal drug exposure exerts a sustained inhibitory effect on virus production or virion infectivity, even after their migration to deeper tissues.

Previous *in vitro* evidence provides support for such a ‘memory effect’, in that pretreatment of chronically infected cells with the non-nucleoside reverse transcriptase inhibitor (NNRTI) UC781 results in the release of attenuated virus [14]. Therefore, here we investigated whether other microbicide candidates exert a similar effect on CAV. To this end, HIV-infected peripheral blood mononuclear cells (PBMCs) were used as a surrogate for migrating seminal leucocytes and treated with antiretrovirals (ARVs) from different classes. Subsequently, extracellular compound and CFV were removed to mimic escape from microbicide exposure. Next, the amount of virus produced from these cells and its relative infectivity were assessed.

Among the test compounds was the CD4-binding site inhibitor M48U1, which inhibits the gp120-CD4 interaction in the nanomolar range by targeting the highly conserved and vulnerable Phe43-cavity in the HIV envelope [15,16], and which showed nearly complete protection in Cynomolgus macaques when applied as a vaginal gel [17].

### Most ARVs do not inhibit virus production by infected cells

PHA/IL-2 stimulated PBMCs were infected with 2 x 10^-3^ multiplicity of infection (MOI) of the CCR5-tropic subtype B strain Bal for three days and subsequently washed extensively to remove the inoculum (Figure 1A). Next, cells were incubated for 24 hours with ARVs from different classes at 100x EC50 concentrations for each compound (Table 1). The virions produced from these cultures were then quantified in quadruplicate by a Gag p24 capture ELISA [18]. Interestingly, pretreatment with most ARVs did not inhibit virus production by infected cells as compared to the untreated control cultures (Figure 1B). However, in the supernatant of cultures treated with the protease inhibitors (PIs) lopinavir and saquinavir, a significantly lower (i.e., 0.5-1 log) Gag p24 concentration was observed, likely as a result of unprocessed Gag precursor.

**Table 1:** Overview of antiretroviral compounds

**Inhibitor**	**Target**	**EC50 (nM)**^**a**^
**UC781**	Reverse transcriptase	35
**Dapivirine (TMC120)**	Reverse transcriptase	5
**Tenofovir (TFV)**	Reverse transcriptase	4800
**Saquinavir (SQV)**	Protease	35
**Lopinavir (LPV)**	Protease	40
**Raltegravir (RLT)**	Integrase	18
**Maraviroc (MRV)**	CCR5	88
**Enfuvirtide (T20)**	Gp41	35
**C34-cholesterol (C34-chol)**	Gp41	7.5
**Griffithsin (GRFT)**	Glycans on gp120	1.5
**M48U1**	CD4 binding site on gp120	35
**Soluble CD4 (sCD4)**	Reverse transcriptase	116

^a^50% effective concentration (EC50) measured in PBMCs against HIV-1 Bal. EC50s were calculated using linear regression analysis on data of one experiment with 6 replicates.

### Pretreatment with M48U1 affects the infectivity of *de novo* produced virions

Next, we evaluated whether these ARVs affected the infectivity of the virus produced. PBMCs were infected as described above and were washed extensively to remove inhibitor and incubated in fresh medium for a new round of viral replication. After 24 h, supernatant was harvested, and then assessed for its ability to infect TZM-bl cells using identical viral inocula (based on equal amounts of Gag p24). The obtained viral titers were expressed as a percentage, relative to the untreated control cultures (Figure 1C). Surprisingly, virions produced by PBMCs pre-treated with M48U1, were almost completely defective (relative titer of <1%). A smaller loss in titer was observed after treatment with the PIs saquinavir (relative titer of 47%) and lopinavir (relative titer of 14%), and the carbohydrate binding protein griffithsin (relative titer of 31%). The same samples were assayed in a multi-cycle infectivity assay with PBMCs, with similar results (Figure 1C). Overall, these observations suggest a continued inhibition by some ARVs on *de novo* produced virions, even *after* their removal. To investigate whether this attenuation was further sustained after multiple rounds of viral replication, supernatant containing *de novo* virus was collected from the same cell cultures at 48 h and 72 h and again titrated on TZM-bl cells and PBMCs (Figure 1A and C). For all viruses that showed a reduced titer at 24 h, a clear increase in infectivity was observed over time. Whereas in PI-treated cultures, virion infectivity was rapidly restored to normal levels; the virions produced by griffithsin- and M48U1-treated cultures remained respectively two and four times less infectious than virions from the control cultures, as late as 72 hours post-exposure (Figure 1C). To exclude a strain-specific effect of the lab-adapted Bal virus, the experiment was repeated for M48U1 using the more relevant transmitted/founder (T/F) viruses REJO and THRO (subtype B). Similar results were obtained with both viruses, showing a severely reduced relative titer at 24 h (3.7% and 3.1%, respectively) that restores to normal infectivity levels over time (Figure 1D).

### The memory effect of M48U1 depends on direct interaction between M48U1 and gp120

To assess the role of M48U1:gp120 interaction in relation to the sustained reduction in infectivity, we used a miniCD4 resistant, but CD4 receptor binding competent mutant virus bearing the S375R mutation (BalS375R). This mutation is located next to the CD4 binding loop and disrupts the molecular interaction of M48U1 with gp120 [18]. Similar to the previous experiments, PBMCs were infected with wild type (WT), and mutant HIV-1 Bal, subsequently treated with a variety of ARVs and washed 24 h later. As before, cultures were then left to produce new virions in the absence of additional ARV pressure. For most ARVs no significant differences in WT or mutant virus production were found after ARV treatment. However, contrasting the low infectious titer (<1%) of WT virus after M48U1 treatment, a normal titer (i.e., 100%) was found for the S375R mutant virus (Figure 1E). This result clearly indicates that the gp120:M48U1 interaction is a prerequisite for the memory effect observed with M48U1. As CFV is removed after treatment, the most logical target for M48U1 is the functional envelope proteins (Env) on infected cell surfaces.

### M48U1 induces gp120 shedding

**Figure 2:**
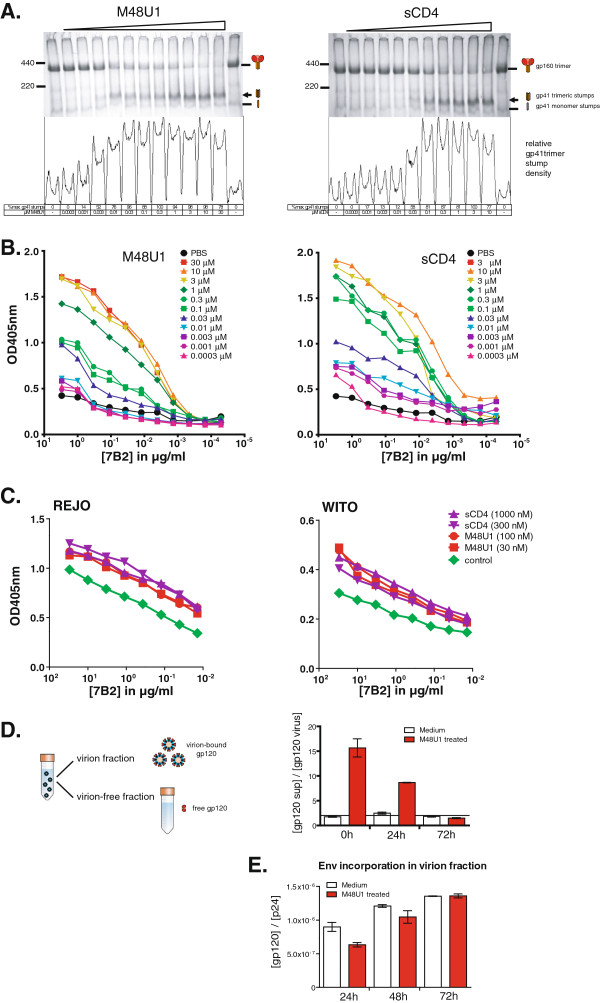
**M48U1 induces gp120 shedding. A)** “Trimer VLPs” (E168K+N189A mutant) expressing trimeric Env of the subtype B virus JR-FL were treated with graded doses of M48U1 or sCD4. Shedding was then assessed using BN-PAGE-Western blot analysis of VLP Env. Below the gel, the density of trimeric gp41 stumps (indicated by an arrow on the gel) is shown, as determined using ImageJ densitometry software (NIH, Bethesda, USA, http://imagej.nih.gov/ij/). Second, we coated the same trimer VLPs of JR-FL **B)** or the subtype B transmitted/founder viruses REJO and WITO (**C**) on an ELISA plate at 20x the concentration present in transfection supernatants, treated then with graded doses of M48U1 or sCD4, as indicated and then measured ELISA binding by mAb 7B2 that reacts with a cryptic epitope of gp41 that only becomes exposed upon the loss of gp120 from native trimers (i.e. gp120 shedding). **D)** Finally, virus particles were isolated with magnetic CD44 microbeads from PBMC culture supernatant at 0 h, 24 h and 72 h after M48U1 exposure. Gp120 was then quantified in virion and virion-free fractions using a D7324-based gp120-ELISA. Shedding was expressed as a ratio of free gp120 in the supernatant and intact Env in the virion fraction and compared to the untreated control cultures (i.e., medium). Values are the means +/− SEM of two independent measurements. **E)** Env incorporation in the virion fraction was determined by quantifying both the gp120 and p24 content in ELISA and plotting the gp120/p24 ratio.

CD4 engagement can result in the spontaneous loss of gp120, without infection, resulting in defective gp41 stumps. To investigate whether gp120 shedding could explain the memory effect observed for M48U1, pseudovirion virus-like particles (VLPs) expressing trimeric Env of the subtype B virus JR-FL were treated with graded doses of sCD4 or M48U1. Two assays were then used to measure gp120 shedding. In the first assay, VLPs were treated with sCD4 or M48U1, washed, then Env was extracted from particles and resolved by BN-PAGE/Western blot, as described elsewhere [19]. In this assay, gp120 shedding was indicated by a loss of intact native Env trimer coupled with an increase in gp41 stumps that are left behind after gp120 shedding. In a second assay, VLPs were treated with sCD4 or M48U1, washed, then coated on ELISA wells and assayed for binding of mAb 7B2, which reacts with the immunodominant cluster I epitope of gp41 that is exposed on the gp41 stumps exposed after gp120 shedding. In both of these assays, we used JR-FL E168K+N189A trimer VLPs generated by protease digestion to eliminate non-functional Env from particle surfaces, as described previously [19]. By eliminating any gp41 stumps present before drug treatment, this enhances the ability to detect new gp41 stumps that appear as a result of drug-induced shedding. In both assays, M48U1 caused shedding: in proportion to the concentration of M48U1 used, there was a loss in native trimer staining coupled with an increase in gp41 stumps in BN-PAGE (Figure 2A) and a quantitative increase in 7B2 binding by ELISA (Figure 2B). Moreover, by the BN-PAGE assay, 50% shedding was induced at approximately 10-fold lower concentrations of M48U1 than of sCD4. This is reflected by the lower EC50 of M48U1 (450nM) compared to sCD4 (900nM) in neutralization assays against JR-FL on PBMCs (data not shown). Consistent with the data on JR-FL, treatment of two T/F viruses (REJO and WITO) with sCD4 and M48U1 also resulted in an increased binding of the 7B2 mAb (Figure 2C). Together, these results show that M48U1 potently induces gp120 shedding.

### Memory effect of M48U1 is linked to gp120 shedding

We next evaluated whether gp120 shedding underlies the observed memory effect of M48U1. If this were the case, treatment of HIV-infected cells with sCD4 should also decrease the infectivity of *de novo* produced virions, as seen with M48U1 in Figure 1C. Indeed, like M48U1, we found that virus produced from infected PBMCs pretreated with sCD4 was fivefold less infectious (~22%) than the control virus (data not shown). Furthermore, if M48U1 induces gp120 shedding in the treated cell cultures, this should be reflected by an increase in gp120 in the virion-depleted supernatant and a reciprocal decrease in virion-associated gp120. Therefore, virus particles were separated from culture supernatants, using magnetic anti-CD44 microbeads (μMACS™ VitalVirus isolation kit, Miltenyi Biotec). We then used an ELISA to quantify gp120 in both fractions. Interestingly, at the earliest time point, gp120 was far more abundant in the virion-depleted supernatant of M48U1 treated cultures than of the control cultures (Figure 2D). The ratio of virion-free and virion-bound gp120 then gradually decreased to reach equilibrium levels 72 h post treatment, coincident with the time-dependent recovery of virus infectivity (Figure 2D). Finally, if M48U1 induces gp120 shedding from the Env spikes embedded in the cell membrane of infected PBMCs, this would predict a higher incorporation of functional Env molecules in virions produced from untreated PBMCs compared to M48U1-treated PBMCs. Using the same method described above, we determined the gp120 and p24 concentration in the virion fraction 24 h, 48 h, and 72 h after M48U1 treatment. Functional Env incorporation was calculated as the ratio of gp120 vs. p24. As shown in Figure 2E, the results suggest that virions produced from infected PBMCs after removal of M48U1 have a significantly lower degree of functional Env incorporation than control virions produced by untreated PBMCs. Once again, and coinciding with the observed time-dependent recovery of virus infectivity, Env incorporation is gradually restored to control levels at 48 h and 72 h.

### Implications of M48U1 in preventing mucosal HIV transmission

Several studies have shown that infected seminal leucocytes can traverse intact vaginal epithelia to reach the submucosa and even the draining lymph nodes [2,4,7]. Although this might not be the dominant route of infection in natural HIV transmission, in the context of microbicide use, CAV might escape high drug concentrations in the vaginal lumen, by migration across the epithelium that lines the female genital tract. However, whether the initial microbicide exposure can affect virus budding from these migrating seminal leucocytes remains unclear. Therefore, we here evaluated the effect of various ARVs on the virus produced after treatment and subsequent washing of infected PBMCs. Most ARVs had no impact on virus production or virion infectivity. However, surprisingly, infected cells exposed to the CD4 mimetic M48U1 produced largely defective viral particles during the first 24 hours after treatment. Virus infectivity was gradually restored at later time points. These results were unexpected because the candidate microbicide M48U1 acts early in the viral lifecycle by preventing HIV entry into host cells through competition with the CD4 receptor. As the infected PBMCs were washed extensively to remove M48U1 and CFV after initial drug exposure, this ‘memory’ effect was unlikely to be due to CFV already neutralized by M48U1 or unbound M48U1 in the supernatant.

We hypothesized that M48U1 associates with gp120 molecules expressed on the cell membrane of infected cells prior to viral budding. If M48U1 dissociates very slowly from its target and is retained after washing, this could cause the production of defective virions. However, if this were the case, one would expect a similar attenuation effect for the tight-binding RT inhibitors UC781 and TMC120. Previous studies have shown that pretreatment of infected cells with UC781 results in a fivefold reduction in the infectivity of *de novo* produced virus, which was explained by the membrane compartment hypothesis [14,20]. In this hypothesis NNRTIs, which are sequestered in the cell plasma membrane due to their hydrophobic nature, would be incorporated into the membrane of nascent budding virions. The tight binding to RT would subsequently trap the NNRTI within the virions, rendering them defective. Although different cells (PBMCs versus H9+ cells) and a different read-out of infectivity (Gag p24 and firefly luciferase versus syncytium formation) were used, our results revealed only a very small memory effect for UC781 (relative titer of 75% on TZM-bl cells). This suggests that the NNRTIs in our assay were removed from the PBMC cultures, making it unlikely that the hydrophilic M48U1, which binds to the gp120 molecules in the plasma membrane, would be retained on its target. Hence, another mechanism must explain the sustained M48U1 activity.

**Figure 3:**
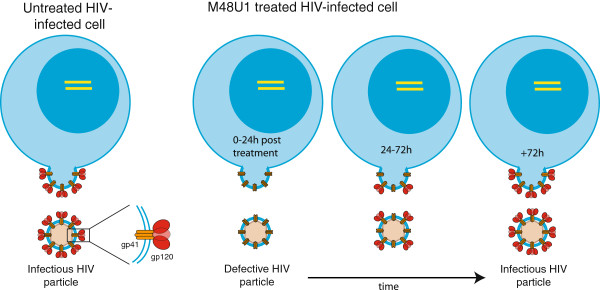
**Proposed model explaining the memory effect of M48U1 through gp120 shedding.** M48U1 induces shedding of gp120 from HIV envelope proteins embedded in the membrane of infected cells prior to budding. This results in the production of defective virions in absence of drug during the first 24 h post treatment. Continuous recruitment of new envelope proteins from the endoplasmic reticulum to the cell membrane explains why infectivity is restored over-time.

More than twenty years ago, soluble CD4 was shown to disrupt the non-covalent association of the gp120 and gp41 envelope glycoproteins when used at high concentrations, rendering virions noninfectious [21,22]. Recently, gp120 shedding was also observed to be induced by membrane-proximal external region (MPER)-specific antibodies 2F5 and 4E10 [23]. Although the antiviral effect of M48U1 is mainly caused by competitive inhibition, gp120 shedding might occur at high M48U1 concentrations and hence explain the prolonged inhibitory effect. If this is true, then the continuous recruitment of freshly synthesized HIV envelope proteins from the endoplasmic reticulum to the cell membrane would explain why infectivity is restored over-time (see model in Figure 3). Here, we provided 4 separate pieces of evidence to support this hypothesis. First, by BN-PAGE, we saw that treatment of VLPs with M48U1 led to an increase in gp41 stumps coupled with a decrease in native Env trimer (Figure 2A). Second, M48U1-induced exposure of the cryptic 7B2 epitope on gp41 stumps on VLP surfaces is also consistent with gp120 shedding (Figure 2B). Third, the larger proportion of gp120 detected in the virus-depleted supernatant of M48U1 treated cultures is again consistent with gp120 shedding (Figure 2C). Finally, we showed that the recovery of virus infectivity coincides with an increased incorporation of functional Env molecules in the budding viruses (Figure 2E). Together, these observations strongly suggest that M48U1 induces gp120 shedding at the cell membrane, resulting in defective nascent virions.

From a microbicide perspective, entry inhibitors with a memory inhibitory effect on CAV are desirable. If infected seminal leucocytes can escape microbicide in the vagina by crossing the mucosal barrier, they could establish a founder population of infected target cells that then expands locally using the influx of new target cells recruited through outside-in signaling [24]. Low tissue concentrations of microbicide are of specific concern when entry inhibitors are used because their hydrophilic nature might impede compound accumulation in the (sub) mucosa. However, if the virus budding from these migrating leucocytes is rendered defective by prior microbicide exposure in the vagina, a window of opportunity would be provided to eliminate these invading cells before local infection is established.

Aside from M48U1, we also observed a decline in infectivity of virus budding from cells that were pre-treated with the PIs lopinavir and saquinavir or the gp120-glycan binder griffithsin, although these effects were modest compared to that of M48U1. The memory inhibitory effect of the PIs most likely results from immature virus particles that are still budding off in the first hours after PI removal. This is supported by the rapid restoration of virion infectivity at later time points. Interestingly, virions from the griffithsin-treated cultures did not completely regain their infectivity 72 h after removal of the ARV. Although the observed memory inhibitory effect of griffithsin remains the subject of ongoing research, it is not due to gp120 shedding (data not shown), indicating that, even after extensive washing, at least some griffithsin is retained within the treated cell culture thereby confirming recent work of Kouokam *et al.*[25].

Although current clinical trials are mainly testing reverse transcriptase inhibitors as microbicides, there is increasing interest in combining different ARV classes into combination microbicides to increase efficacy and to avoid cross-resistance with first-line therapy. Despite being a miniproteïn, the entry inhibitor M48U1 is easy to produce, is poorly immunogenic and does not induce anti-CD4 antibodies in vivo [26]. Together with its small size (27 amino acids), stable conformation in denaturing conditions (i.e., acidic pH and high temperatures) and relative resistance towards proteases [15], M48U1 thus has a favorable profile as potential microbicide. This was confirmed in a recent trial with a M48U1-loaded gel showing almost complete protection (5 out of 6 animals) after vaginal challenge in macaques [17]. The relative ease at which HIV escapes inhibition by antibodies or small molecules that target the entry process, including M48U1 argues for its use in a combination microbicide [18].

In conclusion, in this study we report for the first time that a highly potent CD4 mimetic HIV entry inhibitor, M48U1, induces gp120 shedding at high compound concentrations resulting in the production of defective nascent virions from infected primary cells up to 72 h after their exposure to the drug. This memory effect adds to the already interesting properties of M48U1 as a potential candidate for development into a combination microbicide.

## Competing interests

The authors declare no competing financial interests.

## Authors’ contributions

PS and KG were involved in the study design, the experimental work and drafting the manuscript. TT, ETC. and JMB performed the envelope shedding experiments. LM produced M48U1 and performed the gp120 ELISA experiments. GV was involved in drafting the manuscript. JMB and KKA wrote the final manuscript. KKA is responsible for the overall study design. All authors read and approved the final manuscript.
